# Supplementary material for: Cavity-QED-controlled two-dimensional Moiré excitons without twisting
Source: Nat Commun. 2025 Dec 24;17:157. doi: 10.1038/s41467-025-67570-2 (PMC12774878; doi:10.1038/s41467-025-67570-2)
Supplement: Supplementary file 1 — Supplementary Information [file 41467_2025_67570_MOESM1_ESM.pdf]

Supplementary information: Cavity-QED-controlled two-dimensional Moiré Excitons without twisting

Francesco Troisi,[1,*] Hannes Hübener,[1,†] Angel Rubio,[1,2,‡] and Simone Latini[1,3,§]

[1] Max Planck Institute for the Structure and Dynamics of Matter and Center for Free-Electron Laser Science, Luruper Chaussee 149, 22761, Hamburg, Germany

[2] Initiative for Computational Catalysis (ICC), The Flatiron Institute, 162 Fifth Avenue, New York, NY 10010, United States

[3] Department of Physics, Technical University of Denmark, 2800 Kgs. Lyngby, Denmark

I. QED HAMILTONIAN DERIVATION

This section contains the derivation of the QED Hamiltonian. We start by formulating it with electronic creation and annihilation operators, based on previous works [1, 2]. Subsequently, we change the basis, firstly to an electron-hole pair basis and finally to the exciton basis [1]. Finally, we show that by doing so the Moiré potential and the bilinear coupling for finite $\bar{\boldsymbol{q}}$ cavities enter the QED Hamiltonian in the same term.

A. QED Hamiltonian with electronic creation and annihilation operators

This work studies Moiré excitons in type-II $MoSe_2/WSe_2$ hetero-structure in optical cavities. Refer to Fig. 1 of the main text for its representation. The two layers are separated by a dielectric medium. Let us call l the index of the layer. Each TMD is represented with a set of k-points and a set of valence and conduction band states. An electron in one of the valence states of either layer is allowed to transition to any conduction state of the same or the other layer, resulting in an intra-layer or inter-layer exciton, respectively.
The uncoupled matter Hamiltonian of the system $\hat{H}_M$, which describes the hetero-structure, can be formulated as the sum of three different terms, the free particle, the Moiré and the Coulomb potential. Using the electronic creation and annihilation operators, the Hamiltonian reads:

$$\begin{aligned}\hat{H}_M = &\sum_{l,i,\boldsymbol{k}}^{C} \varepsilon_{l,i,\boldsymbol{k}} \hat{c}^{\dagger}_{l,i,\boldsymbol{k}} \hat{c}_{l,i,\boldsymbol{k}} + \sum_{l,i,\boldsymbol{k}}^{V} \varepsilon_{l,i,\boldsymbol{k}} \hat{v}^{\dagger}_{l,i,\boldsymbol{k}} \hat{v}_{l,i,\boldsymbol{k}} + \sum_{ll'} \sum_{ij}^{C} \sum_{\boldsymbol{kq}} V^{C}_{l}(\boldsymbol{q})\, \hat{c}^{\dagger}_{l,i,\boldsymbol{k+q}} \hat{c}_{l',j,\boldsymbol{k}} + \\ &\sum_{ll'} \sum_{ij}^{V} \sum_{\boldsymbol{kq}} V^{V}_{l}(\boldsymbol{q})\, \hat{v}^{\dagger}_{l,i,\boldsymbol{k+q}} \hat{v}_{l',j,\boldsymbol{k}} + \sum_{ll'} \sum_{ij} \sum_{\boldsymbol{kk'q}} \mathcal{W}^{ll'}_{\boldsymbol{q}} \hat{c}^{\dagger}_{l,i,\boldsymbol{k+q}} \hat{v}^{\dagger}_{l',j,\boldsymbol{k'-q}} \hat{v}_{l',j,\boldsymbol{k'}} \hat{c}_{l,i,\boldsymbol{k}} + h.c.\end{aligned} \tag{1}$$

where i, j are band indexes which span over conduction (C) or valence (V) band states, l, l' are layer indexes, $\boldsymbol{k}, \boldsymbol{k'}, \boldsymbol{q}$ are k-points indexes. The operators $\hat{c}^{\dagger}, \hat{c}$ ($\hat{v}^{\dagger}, \hat{v}$) create or annihilate an electron in the conduction (valence) band. $\mathcal{W}^{ll'}_{\boldsymbol{q}}$ is the matrix element of the Coulomb potential [3]. $V_l(\boldsymbol{q})$ is the Moiré potential coefficient [1]:

$$V_l(\boldsymbol{q}) = v_l \sum_{n=0}^{2} e^{iC_3^n\left(\boldsymbol{G}^{\boldsymbol{0}}_{l} + \boldsymbol{G}^{\boldsymbol{0}}_{l'}\right)) \cdot \boldsymbol{D}_l/2} \delta_{\boldsymbol{q}, C_3^n\left(\boldsymbol{G}^{\boldsymbol{0}}_{l} - \boldsymbol{G}^{\boldsymbol{0}}_{l'}\right))} ; v_l = \alpha_l + e^{2\pi i \sigma_{l'}/3} \beta_l \tag{2}$$

The values of α_l, β_l are taken from Table 1 of the Supporting Information of Ref. [1], while $\sigma_l = 1$ for all layers (R-type stacking).

We describe the uncoupled light system with an Hamiltonian consisting of a set of effective harmonic oscillators. As for the light-matter coupling, we perform the canonical momentum substitution $\hat{\boldsymbol{p}} \rightarrow \hat{\boldsymbol{p}} + \hat{\boldsymbol{A}}$ on the uncoupled matter Hamiltonian, obtaining the second-quantized Pauli-Fierz Hamiltonian [2]. The full QED Hamiltonian can then be formulated as follows [2]:

---

* francesco.troisi@mpsd.mpg.de
† hannes.huebener@mpsd.mpg.de
‡ angel.rubio@mpsd.mpg.de
§ simola@dtu.dk

$$\hat{H}_{QED} = \hat{H}_M + \hat{H}_b + \hat{H}_d + \sum_{\bar{\boldsymbol{q}},\lambda} \omega_{\bar{\boldsymbol{q}}} \left(\hat{a}^\dagger_{\bar{\boldsymbol{q}},\lambda} \hat{a}_{\bar{\boldsymbol{q}},\lambda} + \frac{1}{2} \right) \tag{3}$$

where λ is the polarization of the mode and $\hat{a}$ ($\hat{a}^\dagger$) is the annihilation (creation) operator for the mode $|\bar{\boldsymbol{q}}, \lambda\rangle$. $\omega_{\bar{\boldsymbol{q}}}$ is the energy of the mode $\bar{\boldsymbol{q}}$. Moreover, we define the renormalized light-matter coupling constant as $\tilde{A}_{0,\bar{\boldsymbol{q}}} = \frac{A_{0,\bar{\boldsymbol{q}}}}{\sqrt{V_{\text{eff},\bar{\boldsymbol{q}}}}}$ ($A_{0,\bar{\boldsymbol{q}}}$ is the coupling strength of the mode $\bar{\boldsymbol{q}}$ and $V_{\text{eff},\bar{\boldsymbol{q}}}$ is the effective mode volume). $\hat{H}_b$ and $\hat{H}_d$, the bilinear and diamagnetic Hamiltonians, are:

$$\hat{H}_b = \sum_{\lambda} \sum_{\boldsymbol{k},\bar{\boldsymbol{q}}} \tilde{A}_{0,\bar{\boldsymbol{q}}} \left[\sum_{ij}^{C} p^{\lambda}_{ij,\boldsymbol{k}+\bar{\boldsymbol{q}},\boldsymbol{k}} \hat{c}^\dagger_{i,\boldsymbol{k}+\bar{\boldsymbol{q}}} \hat{c}_{j,\boldsymbol{k}} + \sum_{i,j}^{V} p^{\lambda}_{ij,\boldsymbol{k}+\bar{\boldsymbol{q}},\boldsymbol{k}} \hat{v}^\dagger_{i,\boldsymbol{k}+\bar{\boldsymbol{q}}} \hat{v}_{j,\boldsymbol{k}} + \sum_{i\in C, j\in V} p^{\lambda}_{ij,\boldsymbol{k}+\bar{\boldsymbol{q}},\boldsymbol{k}} \hat{c}^\dagger_{i,\boldsymbol{k}+\bar{\boldsymbol{q}}} \hat{v}_{j,\boldsymbol{k}} \right] \left(\hat{a}^\dagger_{\bar{\boldsymbol{q}},\lambda} + \hat{a}_{-\bar{\boldsymbol{q}},\lambda} \right) \tag{4}$$

$$\hat{H}_d = \sum_{\lambda\lambda'} \sum_{\boldsymbol{k}\bar{\boldsymbol{q}}\bar{\boldsymbol{q}}'} \frac{\tilde{A}_{0,\bar{\boldsymbol{q}}} \tilde{A}_{0,\bar{\boldsymbol{q}}'}}{2} \left(\hat{a}^\dagger_{\bar{\boldsymbol{q}},\lambda} + \hat{a}_{-\bar{\boldsymbol{q}},\lambda} \right) \left(\hat{a}^\dagger_{\bar{\boldsymbol{q}}',\lambda'} + \hat{a}_{-\bar{\boldsymbol{q}}',\lambda'} \right) * \left[\sum_{ij}^{C} s^{\lambda\lambda'}_{ij,\boldsymbol{k}-\bar{\boldsymbol{q}}+\bar{\boldsymbol{q}}'} \hat{c}^\dagger_{i,\boldsymbol{k}+\bar{\boldsymbol{q}}} \hat{c}_{j,\boldsymbol{k}} + \sum_{ij}^{V} s^{\lambda\lambda'}_{ij,\boldsymbol{k}-\bar{\boldsymbol{q}}+\bar{\boldsymbol{q}}'} \hat{v}^\dagger_{i,\boldsymbol{k}-\bar{\boldsymbol{q}}+\bar{\boldsymbol{q}}'} \hat{v}_{j,\boldsymbol{k}} + \sum_{i\in C, j\in V} s^{\lambda\lambda'}_{ij,\boldsymbol{k}-\bar{\boldsymbol{q}}+\bar{\boldsymbol{q}}'} \hat{c}^\dagger_{i,\boldsymbol{k}-\bar{\boldsymbol{q}}+\bar{\boldsymbol{q}}'} \hat{v}_{j,\boldsymbol{k}} \right] \tag{5}$$

where $p^{\lambda}_{ij,\boldsymbol{k}+\bar{\boldsymbol{q}},\boldsymbol{k}}$ is the momentum matrix element and $s^{\lambda\lambda'}_{ij,\boldsymbol{k}-\bar{\boldsymbol{q}}+\bar{\boldsymbol{q}}'}$ is the overlap matrix element [2].

Note that in equations 3-5 the symbol $\bar{\boldsymbol{q}}$ appears as the momentum associated with the photonic mode. This shall not be confused with the $\boldsymbol{q}$ which appears in eq 1-2, which is the reciprocal lattice vector associated with the periodicity of the Moiré potential.

B. QED Hamiltonian in the electron-hole basis

We prefer to use an exciton representation to study the behavior of excitons in such a system, as it allows to directly encode the effect of the Coulomb potential in the formation of such bound quasi-particles. To address this problem, let us define the electron-hole operators and their low-density expansions [1]:

$$\hat{P}^\dagger_{l,i,\boldsymbol{k};l',j,\boldsymbol{k}'} = \hat{c}^\dagger_{l,i,\boldsymbol{k}} \hat{v}_{l',j,\boldsymbol{k}'} \tag{6}$$

$$\hat{P}_{l,i,\boldsymbol{k};l',j,\boldsymbol{k}'} = \hat{v}^\dagger_{l',j,\boldsymbol{k}'} \hat{c}_{l,i,\boldsymbol{k}} \tag{7}$$

$$\hat{c}^\dagger_{l,i,\boldsymbol{k}} \hat{c}_{l',j,\boldsymbol{k}'} \approx \sum_{m,a,\boldsymbol{q}} \hat{P}^\dagger_{l,i,\boldsymbol{k};a,m,\boldsymbol{q}} \hat{P}_{l',j,\boldsymbol{k}';m,a,\boldsymbol{q}} \tag{8}$$

$$\hat{v}^\dagger_{l,i,\boldsymbol{k}} \hat{v}_{l',j,\boldsymbol{k}'} \approx \delta_{ll',ij,\boldsymbol{k}\boldsymbol{k}'} - \sum_{m,a,\boldsymbol{q}} \hat{P}^\dagger_{a,m,\boldsymbol{q};l,i,\boldsymbol{k}} \hat{P}_{m,a,\boldsymbol{q};l',j,\boldsymbol{k}'} \tag{9}$$

In the following, the indexes of the $\hat{P}$ operators will always follow this order: layer index, band index, $\boldsymbol{k}$ point index. This is regardless of the letters used. By using these relations, we can transform the QED Hamiltonian as follows:

$$\hat{H}_f = \sum_{l,i,\boldsymbol{k}}^{V} \varepsilon_{l,i,\boldsymbol{k}} - \sum_{l,j,\boldsymbol{k}}^{V} \varepsilon_{l,j,\boldsymbol{k}} \sum_{r,s,\boldsymbol{p}}^{C} \hat{P}^\dagger_{r,s,\boldsymbol{p};l,j,\boldsymbol{k}} \hat{P}_{r,s,\boldsymbol{p},l,j,\boldsymbol{k}} + \sum_{l,i,\boldsymbol{k}}^{C} \varepsilon_{l,i,\boldsymbol{k}} \sum_{r,s,\boldsymbol{p}}^{V} \hat{P}^\dagger_{l,i,\boldsymbol{k};r,s,\boldsymbol{p}} \hat{P}_{l,i,\boldsymbol{k};r,s,\boldsymbol{p}} \tag{10}$$

$$\hat{H}_m = \sum_{l,j,\boldsymbol{k}}^{V} V_{l,i}(\boldsymbol{0}) - \sum_{ij}^{V} \sum_{ll'} \sum_{\boldsymbol{k}\boldsymbol{q}} V_{ll',ij}(\boldsymbol{q}) \sum_{r,s,\boldsymbol{p}}^{C} \hat{P}^\dagger_{r,s,\boldsymbol{p};l',j,\boldsymbol{k}} \hat{P}_{r,s,\boldsymbol{p};l,i,\boldsymbol{k}+\boldsymbol{q}} + \sum_{ij}^{C} \sum_{ll'} \sum_{\boldsymbol{k}\boldsymbol{q}} V_{ll',ij}(\boldsymbol{q}) \sum_{r,s,\boldsymbol{p}}^{V} \hat{P}^\dagger_{l,i,\boldsymbol{k}+\boldsymbol{q};r,s,\boldsymbol{p}} \hat{P}_{l,i,\boldsymbol{k};r,s,\boldsymbol{p}} \tag{11}$$

$$\hat{H}_c = \sum_{l,i,\boldsymbol{k}}^{C} \mathcal{W}^{ll'}_{\boldsymbol{0}} \hat{c}^\dagger_{l,i,\boldsymbol{k}} \hat{c}_{l,i,\boldsymbol{k}} - \sum_{l,i,\boldsymbol{k}}^{C} \sum_{l',j,\boldsymbol{k}'}^{V} \sum_{\boldsymbol{q}} \mathcal{W}^{ll'}_{\boldsymbol{q}} \hat{P}^\dagger_{l,i,\boldsymbol{k}+\boldsymbol{q};l',j,\boldsymbol{k}'} \hat{P}_{l,i,\boldsymbol{k}+\boldsymbol{q};l',j,\boldsymbol{k}'-\boldsymbol{q}} \tag{12}$$

$$\hat{H}_{bil} = \sum_{ll'} \sum_{\boldsymbol{k},\bar{\boldsymbol{q}},\lambda} \tilde{A}_{0,\bar{\boldsymbol{q}}} \left[\sum_{ij}^{V} p^{\lambda}_{ij,ll',\boldsymbol{k}+\bar{\boldsymbol{q}},\boldsymbol{k}} \delta_{ij,ll',\bar{\boldsymbol{q}}=\boldsymbol{0}} + \sum_{ij}^{C} \sum_{r,s,\boldsymbol{p}}^{V} p^{\lambda}_{ij,\boldsymbol{k}+\bar{\boldsymbol{q}},\boldsymbol{k}} \hat{P}^{\dagger}_{l,i,\boldsymbol{k}+\bar{\boldsymbol{q}};r,s,\boldsymbol{p}} \hat{P}_{l',j,\boldsymbol{k};r,s,\boldsymbol{p}} - \right.$$
$$\left. \sum_{ij}^{V} \sum_{r,s,\boldsymbol{p}}^{C} p^{\lambda}_{ij,\boldsymbol{k}+\bar{\boldsymbol{q}},\boldsymbol{k}} \hat{P}^{\dagger}_{r,s,\boldsymbol{p};l',j,\bar{\boldsymbol{q}}} \hat{P}_{r,s,\boldsymbol{p};l,i,\boldsymbol{k}+\bar{\boldsymbol{q}}} + \sum_{i\in C,j\in V} p^{\lambda}_{ij,\boldsymbol{k}+\bar{\boldsymbol{q}},\boldsymbol{k}} \hat{P}^{\dagger}_{l,i,\boldsymbol{k}+\bar{\boldsymbol{q}};l',j,\boldsymbol{k}} + h.c. \right] \left(\hat{a}^{\dagger}_{\bar{\boldsymbol{q}},\lambda} + \hat{a}_{-\bar{\boldsymbol{q}},\lambda} \right) \tag{13}$$

$$\hat{H}_{dia} = \sum_{\lambda\lambda'} \sum_{ll'} \sum_{\boldsymbol{k},\bar{\boldsymbol{q}},\bar{\boldsymbol{q}}'} \frac{\tilde{A}_{0,\bar{\boldsymbol{q}}} \tilde{A}_{0,\bar{\boldsymbol{q}}'}}{2} \left[\sum_{ij}^{V} s^{\lambda\lambda'}_{ij,ll',\boldsymbol{k}-\bar{\boldsymbol{q}}+\bar{\boldsymbol{q}}'} \delta_{ij,ll',\bar{\boldsymbol{q}}=\bar{\boldsymbol{q}}'=\boldsymbol{0}} + \sum_{ij}^{C} \sum_{r,s,\boldsymbol{p}}^{V} s^{\lambda\lambda'}_{ij,\boldsymbol{k}-\bar{\boldsymbol{q}}+\bar{\boldsymbol{q}}'} \hat{P}^{\dagger}_{l,i,\boldsymbol{k}-\bar{\boldsymbol{q}}+\bar{\boldsymbol{q}}';r,s,\boldsymbol{p}} \hat{P}_{l',j,\boldsymbol{k};r,s,\boldsymbol{p}} - \right.$$
$$\left. \sum_{ij}^{V} \sum_{r,s,\boldsymbol{p}}^{C} s^{\lambda\lambda'}_{ij,\boldsymbol{k}-\bar{\boldsymbol{q}}+\bar{\boldsymbol{q}}'} \hat{P}^{\dagger}_{r,s,\boldsymbol{p};l',j,\boldsymbol{k}} \hat{P}_{r,s,\boldsymbol{p};l,i,\boldsymbol{k}-\bar{\boldsymbol{q}}+\bar{\boldsymbol{q}}'} + \sum_{i\in C,j\in V} s^{\lambda\lambda'}_{ij,\boldsymbol{k}-\bar{\boldsymbol{q}}+\bar{\boldsymbol{q}}'} \hat{P}^{\dagger}_{l,i,\boldsymbol{k}-\bar{\boldsymbol{q}}+\bar{\boldsymbol{q}}';l',j,\boldsymbol{k}} \right] \left(\hat{a}^{\dagger}_{\bar{\boldsymbol{q}},\lambda} + \hat{a}_{-\bar{\boldsymbol{q}},\lambda} \right) \left(\hat{a}^{\dagger}_{\bar{\boldsymbol{q}}',\lambda'} + \hat{a}_{-\bar{\boldsymbol{q}}',\lambda'} \right) \tag{14}$$

C. QED Hamiltonian in the excitonic basis

The basis used in the previous section can be further optimized for the present problem. For this purpose, we introduce the bound excitons operators $\hat{X}^{l,l'}_{\boldsymbol{Q}}$, where $\boldsymbol{Q}$ is the center of mass momentum of the exciton, such that:

$$\hat{P}^{\dagger}_{l,i,\boldsymbol{k};l',j,\boldsymbol{k}'} = \sum_{\nu} \hat{X}^{\nu\dagger}_{ll',\boldsymbol{k}-\boldsymbol{k}'} \psi^{\nu}_{ll'} \left(\alpha_{ll'} \boldsymbol{k}' + \beta_{ll'} \boldsymbol{k} \right) \tag{15}$$

where $\alpha_{ll'}$ and $\beta_{ll'}$ are the reduced electron and hole masses [1], ν is the excitonic state (i.e. $\nu = 1s, 2s...$) and ψ is the excitonic wave function from the Wannier equation [4]:

$$\frac{\hbar^2 k^2}{2m^r_{ll'}} \psi^{\nu}_{ll'}(\boldsymbol{k}) - \sum_{\boldsymbol{q}} \mathcal{W}^{ll'}_{\boldsymbol{q}} \psi^{\nu}_{ll'}(\boldsymbol{k}+\boldsymbol{q}) = \hat{E}^{\nu}_{ll'} \psi^{\nu}_{ll'}(\boldsymbol{k}) \tag{16}$$

In the following derivations, we will consider the ν index explicitly. However, in the simulation we limit to $\nu = 1s$. In the previous equation, $\mathcal{W}^{ll'}_{\boldsymbol{q}}$ is the Coulomb potential defined in 1 and $\hat{E}^{\nu}_{ll'}$ is the binding energy. Note that the Wannier equation gives the excitonic states for a specific combination of l, l'. In this work, solving it for $l = l' = 1$ gives the intra-layer excitons for the $MoSe_2$ layer, while solving for $l = l' = 2$ gives the intra-layer excitons for the WSe_2 layer and $l \neq l'$ gives the inter-layer excitons (electron jumping from a valence band state of $MoSe_2$ to the conduction band of WSe_2).

First let us see how the Hamiltonian (eq 10-14) transforms with the introduction of the $\hat{X}$ operators (eq 15). Moreover, we also define $\boldsymbol{Q} = \boldsymbol{k} - \boldsymbol{p}$, which is convenient to simplify the equations. Finally, we neglect the constant terms (i.e. the ones without any operator) in Eq. 10 and Eq. 11, as they only appear in the main diagonal of the matrix. As for the constant term in Eq. 13, we approximate $p^{\lambda}_{ij,ll',\boldsymbol{k}+\bar{\boldsymbol{q}},\boldsymbol{k}} \delta_{ij,ll',\bar{\boldsymbol{q}}=\boldsymbol{0}} = p^{\lambda}_{ii,ll,\boldsymbol{k},\boldsymbol{k}} \approx \frac{\|\boldsymbol{k}\|}{m_h}$ [5].

$$\hat{H}_f = \sum_{\nu} \sum_{l,i,\boldsymbol{Q}}^{C} \sum_{r,s,\boldsymbol{k}}^{V} \varepsilon_{l,i,\boldsymbol{k}} \psi^{\nu}_{lr,is} \left[\alpha_{lr,is} (\boldsymbol{k}-\boldsymbol{Q}) + \beta_{lr,is} \boldsymbol{k} \right] \psi^{\nu*}_{lr,is} \left[\alpha_{lr,is} (\boldsymbol{k}-\boldsymbol{Q}) + \beta_{lr,is} \boldsymbol{k} \right] \hat{X}^{\nu\dagger}_{lr,is,\boldsymbol{Q}} \hat{X}^{\nu}_{lr,is,\boldsymbol{Q}} -$$
$$\sum_{\nu} \sum_{l,j,\boldsymbol{Q}}^{V} \sum_{r,s,\boldsymbol{k}}^{C} \varepsilon_{l,j,\boldsymbol{k}} \psi^{\nu}_{ls,ir} \left[\alpha_{lr,is} \boldsymbol{k} + \beta_{lr,is} (\boldsymbol{k}-\boldsymbol{Q}) \right] \psi^{\nu*}_{ls,ir} \left[\alpha_{lr,is} \boldsymbol{k} + \beta_{lr,is} (\boldsymbol{k}-\boldsymbol{Q}) \right] \hat{X}^{\nu\dagger}_{lr,js,-\boldsymbol{Q}} \hat{X}^{\nu}_{lr,is,-\boldsymbol{Q}} \tag{17}$$

$$\hat{H}_m = \sum_{ll'} \sum_{i\in C,j\in V} \sum_{\boldsymbol{Q},\boldsymbol{q},\nu} \left[\sum_{s}^{C} V^{is}_{ll'}(\boldsymbol{q}) \sum_{r,\boldsymbol{k}} \psi^{\nu}_{lr,ij} \left[\alpha^{ij}_{lr} (\boldsymbol{k}-\boldsymbol{Q}) + \beta^{ij}_{lr} (\boldsymbol{k}+\boldsymbol{q}) \right] \psi^{\nu*}_{l'r,js} \left[\alpha^{js}_{l'r} (\boldsymbol{k}-\boldsymbol{Q}) + \beta^{js}_{l'r} (\boldsymbol{k}) \right] - \right.$$
$$\left. \sum_{s}^{V} V^{js}_{ll'}(\boldsymbol{q}) \sum_{r,\boldsymbol{k}} \psi^{\nu}_{l'r,is} \left[\alpha^{is}_{l'r} (\boldsymbol{k}) + \beta^{is}_{l'r} (\boldsymbol{k}-\boldsymbol{Q}) \right] \psi^{\nu*}_{lr,is} \left[\alpha^{ij}_{lr} (\boldsymbol{k}+\boldsymbol{q}) + \beta^{ij}_{lr} (\boldsymbol{k}-\boldsymbol{Q}) \right] \right] \hat{X}^{\nu\dagger}_{ll',ij,\boldsymbol{Q}+\boldsymbol{q}} \hat{X}^{\nu}_{ll',ij,\boldsymbol{Q}} \tag{18}$$

$$\hat{H}_c = \sum_{l,i,\boldsymbol{Q}}^{C} \sum_{l',j,\boldsymbol{k}}^{V} \sum_{\nu,\boldsymbol{q}} \mathcal{W}_{\boldsymbol{q}}^{ll'} \psi_{ll',ij}^{\nu} \left[\alpha_{ll'}^{ij}\left(\boldsymbol{k}-\boldsymbol{Q}\right)+\beta_{ll'}^{ij}\left(\boldsymbol{k}+\boldsymbol{q}\right)\right] \psi_{ll',ij}^{\nu *} \left[\alpha_{ll'}^{ij}\left(\boldsymbol{k}-\boldsymbol{Q}\right)+\beta_{ll'}^{ij}\left(\boldsymbol{k}+\boldsymbol{q}\right)\right] \hat{X}_{ll',ij,\boldsymbol{Q}+\boldsymbol{q}}^{\nu\dagger} \hat{X}_{ll',ij,\boldsymbol{Q}+\boldsymbol{q}}^{\nu} \tag{19}$$

$$\begin{aligned}\hat{H}_{bil} = \sum_{\lambda,\bar{\boldsymbol{q}}} \tilde{A}_{0,\bar{\boldsymbol{q}}} \sum_{ll'} \sum_{i\in C,j\in V} \sum_{\boldsymbol{Q},\nu} \Bigg\{ \frac{\|\boldsymbol{Q}\|}{m_h} \delta_{ll',ij,\bar{\boldsymbol{q}}=\boldsymbol{0}} + \sum_{\boldsymbol{k}} p_{ij,\boldsymbol{k}+\bar{\boldsymbol{q}},\boldsymbol{k}}^{\lambda} \psi_{ll',ij}^{\nu} \left[\alpha_{ll'}^{ij}\left(\boldsymbol{k}\right)+\beta_{ll'}^{ij}\left(\boldsymbol{k}-\boldsymbol{Q}\right)\right] \hat{X}_{ll',ij,\bar{\boldsymbol{q}}}^{\nu\dagger} + \\ \hat{X}_{ll',ij,\boldsymbol{Q}+\bar{\boldsymbol{q}}}^{\nu\dagger} \hat{X}_{ll',ij,\boldsymbol{Q}}^{\nu} \Bigg[\sum_{r,s,\boldsymbol{k}}^{C} p_{is,\boldsymbol{k}+\bar{\boldsymbol{q}},\boldsymbol{k}}^{\lambda} \psi_{lr,ij}^{\nu} \left[\alpha_{lr}^{ij}\left(\boldsymbol{k}-\boldsymbol{Q}\right)+\beta_{lr}^{ij}\left(\boldsymbol{k}+\bar{\boldsymbol{q}}\right)\right] \psi_{l'r,js}^{\nu *} \left[\alpha_{l'r}^{js}\left(\boldsymbol{k}-\boldsymbol{Q}\right)+\beta_{l'r}^{js}\left(\boldsymbol{k}\right)\right] - \\ \sum_{r,s,\boldsymbol{k}}^{V} p_{js,\boldsymbol{k}+\bar{\boldsymbol{q}},\boldsymbol{k}}^{\lambda} \psi_{l'r,js}^{\nu} \left[\alpha_{l'r}^{js}\left(\boldsymbol{k}\right)+\beta_{l'r}^{js}\left(\boldsymbol{k}-\boldsymbol{Q}\right)\right] \psi_{lr,ij}^{\nu *} \left[\alpha_{lr}^{ij}\left(\boldsymbol{k}+\bar{\boldsymbol{q}}\right)+\beta_{lr}^{ij}\left(\boldsymbol{k}-\boldsymbol{Q}\right)\right] \Bigg] + h.c. \Bigg\} \left(\hat{a}_{\bar{\boldsymbol{q}},\lambda}^{\dagger}+\hat{a}_{-\bar{\boldsymbol{q}},\lambda}\right)\end{aligned} \tag{20}$$

$$\begin{aligned}\hat{H}_{dia} = \sum_{\lambda\lambda'} \sum_{\bar{\boldsymbol{q}},\bar{\boldsymbol{q}}'} \frac{\tilde{A}_{0,\bar{\boldsymbol{q}}}\tilde{A}_{0,\bar{\boldsymbol{q}}'}}{2} \Bigg\{ \sum_{ll'} \sum_{i\in C,j\in V} \sum_{\boldsymbol{Q},\nu} s_{ll,jj,\boldsymbol{Q}}^{\lambda\lambda'} \delta_{ij,ll',\bar{\boldsymbol{q}}=\bar{\boldsymbol{q}}'=\boldsymbol{0}} + \\ \Bigg[\sum_{r,s,\boldsymbol{k}}^{C} s_{is,\boldsymbol{k}-\bar{\boldsymbol{q}}+\bar{\boldsymbol{q}}'}^{\lambda\lambda'} \psi_{lr,ij}^{\nu} \left[\alpha_{lr}^{ij}\left(\boldsymbol{k}-\boldsymbol{Q}\right)+\beta_{lr}^{ij}\left(\boldsymbol{k}-\bar{\boldsymbol{q}}+\bar{\boldsymbol{q}}'\right)\right] \psi_{l'r,js}^{\nu *} \left[\alpha_{l'r}^{js}\left(\boldsymbol{k}-\boldsymbol{Q}\right)+\beta_{l'r}^{js}\left(\boldsymbol{k}\right)\right] - \\ \sum_{r,s,\boldsymbol{k}}^{V} s_{js,\boldsymbol{k}-\bar{\boldsymbol{q}}+\bar{\boldsymbol{q}}'}^{\lambda\lambda'} \psi_{l'r,is}^{\nu} \left[\alpha_{l'r}^{is}\left(\boldsymbol{k}\right)+\beta_{l'r}^{ij}\left(\boldsymbol{k}-\boldsymbol{Q}\right)\right] \psi_{lr,ij}^{\nu *} \left[\alpha_{lr}^{ij}\left(\boldsymbol{k}-\bar{\boldsymbol{q}}+\bar{\boldsymbol{q}}'\right)+\beta_{lr}^{is}\left(\boldsymbol{k}-\boldsymbol{Q}\right)\right] \Bigg] \hat{X}_{ll',ij,\boldsymbol{Q}-\bar{\boldsymbol{q}}+\bar{\boldsymbol{q}}'}^{\nu\dagger} \hat{X}_{ll',ij,\boldsymbol{Q}}^{\nu\dagger} + \\ \sum_{ll'} \sum_{i\in C,j\in V} \sum_{\boldsymbol{k},\nu} s_{ij,\boldsymbol{k}-\bar{\boldsymbol{q}}+\bar{\boldsymbol{q}}'}^{\lambda\lambda'} \psi_{ll',ij}^{\nu} \left[\alpha_{ll'}^{ij}\left(\boldsymbol{k}\right)+\beta_{ll'}^{ij}\left(\boldsymbol{k}-\bar{\boldsymbol{q}}+\bar{\boldsymbol{q}}'\right)\right] \hat{X}_{ll',ij,\bar{\boldsymbol{q}}-\bar{\boldsymbol{q}}'}^{\nu\dagger} + h.c. \Bigg\} \left(\hat{a}_{\bar{\boldsymbol{q}},\lambda}^{\dagger}+\hat{a}_{-\bar{\boldsymbol{q}},\lambda}\right)\left(\hat{a}_{\bar{\boldsymbol{q}}',\lambda'}^{\dagger}+\hat{a}_{-\bar{\boldsymbol{q}}',\lambda'}\right)\end{aligned} \tag{21}$$

After having expressed the Hamiltonian in terms of the operators $\hat{X}$ and of the Wannier wavefunctions ψ, we can further simplify the expression by combining $\hat{H}_f$ and $\hat{H}_c$ using Eq. 16. Then, we can write:

$$\hat{H}_f = \sum_{ll'} \sum_{i\in C,j\in V} \sum_{\nu,\boldsymbol{Q}} \mathcal{E}_{ll',ij,\boldsymbol{Q}}^{\nu} \hat{X}_{ll',ij,\boldsymbol{Q}}^{\nu\dagger} \hat{X}_{ll',ij,\boldsymbol{Q}}^{\nu} \tag{22}$$

As a final step, for the sake of shortening the above expressions, it is convenient to define some common quantities. First, let us define:

$$\Psi_{ll'r,ijs}^{\nu}\left(\boldsymbol{k}',\boldsymbol{k}'',\boldsymbol{k}''',\boldsymbol{k}''''\right) = \psi_{lr,ij}^{\nu}\left[\alpha_{lr}^{ij}\left(\boldsymbol{k}'\right)+\beta_{lr}^{ij}\left(\boldsymbol{k}''\right)\right] \psi_{l'r,is}^{\nu *}\left[\alpha_{lr}^{is}\left(\boldsymbol{k}'''\right)+\beta_{lr}^{is}\left(\boldsymbol{k}''''\right)\right] \tag{23}$$

And subsequently the form factor:

$$\mathcal{F}_{ll',ijs}^{\nu}\left(\boldsymbol{k}',\boldsymbol{k}'',\boldsymbol{k}''',\boldsymbol{k}''''\right) = \sum_{r,\boldsymbol{k}} \Psi_{ll'r,ijs}^{\nu}\left(\boldsymbol{k}',\boldsymbol{k}'',\boldsymbol{k}''',\boldsymbol{k}''''\right) \tag{24}$$

Note that the expression for the form factor is an extension of Eq. 16 of the Supplementary Information of [1]. The two equations become the same if we use only one valence and one conduction band to describe the system (i.e. if we drop the indexes i, j, s). Using Eq. 24 we can now define the Moiré potential prefactor as:

$$\mathcal{M}_{ll',ij,\boldsymbol{Q},\boldsymbol{q}}^{\nu} = \sum_{s}^{C} V_{ll'}^{is}\left(\boldsymbol{q}\right) \mathcal{F}_{ll',ijs}^{\nu}\left(\boldsymbol{k}-\boldsymbol{Q},\boldsymbol{k}+\boldsymbol{q},\boldsymbol{k}-\boldsymbol{Q},\boldsymbol{q}\right) - \sum_{s}^{V} V_{ll'}^{js}\left(\boldsymbol{q}\right) \mathcal{F}_{ll',ijs}^{\nu}\left(\boldsymbol{k},\boldsymbol{k}+\boldsymbol{q},\boldsymbol{k},\boldsymbol{k}-\boldsymbol{Q}\right) \tag{25}$$

After defining this quantity, we can rewrite Eq. 18 as:

$$\hat{H}_m = \sum_{ll'} \sum_{i\in C,j\in V} \sum_{\boldsymbol{Q},\boldsymbol{q},\nu} \mathcal{M}_{ll',ij,\boldsymbol{Q},\boldsymbol{q}}^{\nu} \hat{X}_{ll',ij,\boldsymbol{Q}+\boldsymbol{q}}^{\nu\dagger} \hat{X}_{ll',ij,\boldsymbol{Q}}^{\nu} \tag{26}$$

We can follow a similar strategy for both the bilinear coupling $\hat{H}_{bil}$ and the diamagnetic $\hat{H}_{dia}$ term. For the former we can define:

$$\mathcal{B}^{\nu,\lambda}_{ll',ij,\boldsymbol{Q},\bar{\boldsymbol{q}}} = \sum_{r,\boldsymbol{k}} \left[\sum_{s}^{C} p^{\lambda}_{is,\boldsymbol{k}+\bar{\boldsymbol{q}},\boldsymbol{k}} \Psi^{\nu}_{ll'r,ijs} \left(\boldsymbol{k}-\boldsymbol{Q},\boldsymbol{k}+\bar{\boldsymbol{q}},\boldsymbol{k}-\boldsymbol{Q},\boldsymbol{k}\right) - \sum_{s}^{V} p^{\lambda}_{js,\boldsymbol{k}+\bar{\boldsymbol{q}},\boldsymbol{k}} \Psi^{\nu *}_{ll'r,ijs} \left(\boldsymbol{k},\boldsymbol{k}-\boldsymbol{Q},\boldsymbol{k}+\bar{\boldsymbol{q}},\boldsymbol{k}-\boldsymbol{Q}\right) \right] \tag{27}$$

$$\mathcal{I}^{\nu,\lambda}_{ll',ij,\boldsymbol{Q},\bar{\boldsymbol{q}}} = \sum_{\boldsymbol{k}} p^{\lambda}_{ij,\boldsymbol{k}+\bar{\boldsymbol{q}},\boldsymbol{k}} \psi^{\nu}_{ll',ij} \left[\alpha^{ij}_{ll'} \left(\boldsymbol{k}\right) + \beta^{ij}_{ll'} \left(\boldsymbol{k}-\boldsymbol{Q}\right) \right] \tag{28}$$

While for the diamagnetic term one has that:

$$\mathcal{D}^{\nu,\lambda\lambda'}_{ll',ij,\boldsymbol{Q},\bar{\boldsymbol{q}},\bar{\boldsymbol{q}}'} = \sum_{r,\boldsymbol{k}} \Bigg[\sum_{s}^{C} s^{\lambda\lambda'}_{is,\boldsymbol{k}-\bar{\boldsymbol{q}}+\bar{\boldsymbol{q}}'} \Psi^{\nu}_{ll'r,ijs} \left(\boldsymbol{k}-\boldsymbol{Q},\boldsymbol{k}-\bar{\boldsymbol{q}}+\bar{\boldsymbol{q}}',\boldsymbol{k}-\boldsymbol{Q},\boldsymbol{k}\right) - \sum_{s}^{V} s^{\lambda\lambda'}_{js,\boldsymbol{k}-\bar{\boldsymbol{q}}+\bar{\boldsymbol{q}}'} \Psi^{\nu *}_{ll'r,ijs} \left(\boldsymbol{k},\boldsymbol{k}-\boldsymbol{Q},\boldsymbol{k}-\bar{\boldsymbol{q}}+\bar{\boldsymbol{q}}',\boldsymbol{k}-\boldsymbol{Q}\right) \Bigg] \tag{29}$$

$$\mathcal{S}^{\nu,\lambda\lambda'}_{ll',ij,\bar{\boldsymbol{q}},\bar{\boldsymbol{q}}'} = \sum_{\boldsymbol{k}} s^{\nu,\lambda\lambda'}_{ij,\boldsymbol{k}-\bar{\boldsymbol{q}}+\bar{\boldsymbol{q}}'} \psi^{\nu}_{ll',ij} \left[\alpha^{ij}_{ll'} \left(\boldsymbol{k}\right) + \beta^{ij}_{ll'} \left(\boldsymbol{k}-\bar{\boldsymbol{q}}+\bar{\boldsymbol{q}}'\right) \right] \tag{30}$$

Finally, we can substitute Eq. 27, 28, 29, 30 into Eq. 18, 20, 21 to obtain the full Hamiltonian in the excitonic base. Before writing the final expression, we neglect the terms that appear with a delta in Eq. 20, 21, as the are just a constant. Thus, the final Hamiltonian reads:

$$\begin{aligned}
\hat{H}_{QED} = &\sum_{\bar{\boldsymbol{q}},\lambda} \omega_{\bar{\boldsymbol{q}}} \left(\hat{a}^{\dagger}_{\bar{\boldsymbol{q}},\lambda} \hat{a}_{\bar{\boldsymbol{q}},\lambda} + \frac{1}{2} \right) + \sum_{ll'} \sum_{i\in C, j\in V} \sum_{\nu,\boldsymbol{Q}} \left(\mathcal{E}^{\nu}_{ll',ij,\boldsymbol{Q}} \hat{X}^{\nu\dagger}_{ll',ij,\boldsymbol{Q}} \hat{X}^{\nu}_{ll',ij,\boldsymbol{Q}} + \sum_{\boldsymbol{q}} \mathcal{M}^{\nu}_{ll',ij,\boldsymbol{Q},\boldsymbol{q}} \hat{X}^{\nu\dagger}_{ll',ij,\boldsymbol{Q}+\boldsymbol{q}} \hat{X}^{\nu}_{ll',ij,\boldsymbol{Q}} \right) + \\
&\sum_{\lambda,\bar{\boldsymbol{q}}} \tilde{A}_{0,\bar{\boldsymbol{q}}} \sum_{ll'} \sum_{i\in C, j\in V} \sum_{\boldsymbol{Q},\nu} \left[\mathcal{I}^{\nu,\lambda}_{ll',ij,\boldsymbol{Q},\bar{\boldsymbol{q}}} \hat{X}^{\nu\dagger}_{ll',ij,\bar{\boldsymbol{q}}} + \mathcal{B}^{\nu,\lambda}_{ll',ij,\boldsymbol{Q},\bar{\boldsymbol{q}}} \hat{X}^{\nu\dagger}_{ll',ij,\boldsymbol{Q}+\bar{\boldsymbol{q}}} \hat{X}^{\nu}_{ll',ij,\boldsymbol{Q}} + h.c. \right] \left(\hat{a}^{\dagger}_{\bar{\boldsymbol{q}},\lambda} + \hat{a}_{-\bar{\boldsymbol{q}},\lambda} \right) + \\
&\sum_{\lambda\lambda'} \sum_{\bar{\boldsymbol{q}},\bar{\boldsymbol{q}}'} \frac{\tilde{A}_{0,\bar{\boldsymbol{q}}} \tilde{A}_{0,\bar{\boldsymbol{q}}'}}{2} \sum_{ll'} \sum_{i\in C, j\in V} \sum_{\boldsymbol{Q},\nu} \mathcal{D}^{\nu,\lambda,\lambda'}_{ll',ij,\boldsymbol{Q},\bar{\boldsymbol{q}},\bar{\boldsymbol{q}}'} \hat{X}^{\nu\dagger}_{ll',ij,\boldsymbol{Q}-\bar{\boldsymbol{q}}+\bar{\boldsymbol{q}}'} \hat{X}^{\nu}_{ll',ij,\boldsymbol{Q}} \left(\hat{a}^{\dagger}_{\bar{\boldsymbol{q}},\lambda} + \hat{a}_{-\bar{\boldsymbol{q}},\lambda} \right) \left(\hat{a}^{\dagger}_{\bar{\boldsymbol{q}}',\lambda'} + \hat{a}_{-\bar{\boldsymbol{q}}',\lambda'} \right) + \\
&\sum_{\lambda\lambda'} \sum_{\bar{\boldsymbol{q}},\bar{\boldsymbol{q}}'} \frac{\tilde{A}_{0,\bar{\boldsymbol{q}}} \tilde{A}_{0,\bar{\boldsymbol{q}}'}}{2} \sum_{ll'} \sum_{i\in C, j\in V} \sum_{\boldsymbol{k},\nu} \mathcal{S}^{\nu,\lambda\lambda'}_{ll',ij,\bar{\boldsymbol{q}},\bar{\boldsymbol{q}}'} \hat{X}^{\nu\dagger}_{ll',ij,\bar{\boldsymbol{q}}-\bar{\boldsymbol{q}}'} \left(\hat{a}^{\dagger}_{\bar{\boldsymbol{q}},\lambda} + \hat{a}_{-\bar{\boldsymbol{q}},\lambda} \right) \left(\hat{a}^{\dagger}_{\bar{\boldsymbol{q}}',\lambda'} + \hat{a}_{-\bar{\boldsymbol{q}}',\lambda'} \right) + h.c.
\end{aligned} \tag{31}$$

Note that in the main text we absorb the diamagnetic term into the uncoupled photon Hamiltonian by performing a Bogoliubov transformation [6].

II. OBSERVABLES

This section discusses the formulas for computing the observables shown in the main text. As stated in the Results Section of the main text, we compute both the linear response function $\chi\left(\omega,\Omega_c,\theta\right)$ and the spectral function $\mathcal{S}\left(\omega,\Omega_c,\theta,\boldsymbol{Q}\right)$.

A. Linear response function

The linear response function $\chi\left(\omega,\Omega_c,\theta\right)$ represents the optical response of the system. It is obtained from applying the linear response theory to the polaritonic states [7]. We only calculate the matter part of such a response by tracing out the photons. Hence, we formulate it as:

$$\chi\left(\omega,\Omega_c,\theta\right) = \sum_{I} \frac{\left|\mathcal{M}_{I,0}\right|^2}{\omega - \left(E_I\left(\Omega_c,\theta\right) - E_0\left(\Omega_c,\theta\right)\right) + i\eta} \tag{32}$$

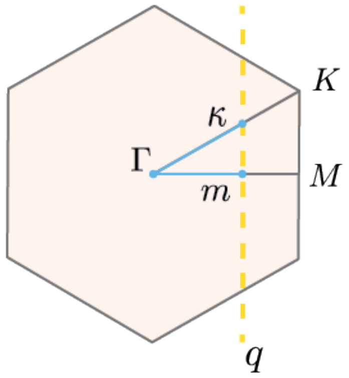

SUPPLEMENTARY FIGURE 1. ***k*-points path**. Representation of the k-points path used to plot the Spectral function in the Moiré Brillouin Zone. The yellow dashed line represents the momentum of the photonic mode $\bar{\boldsymbol{q}}$

where $\mathcal{M}_{I,0} = \langle \Psi_I | \hat{P} \otimes \mathbb{I} \cdot \boldsymbol{e} | \Psi_0 \rangle$ is the transition matrix element between the polaritonic ground state Ψ_0 and an excited state Ψ_I. $\mathbb{I}$ represents the identity operator for the photonic part. η is a small artificial broadening, ω is the energy of the probe field, and $E_I(\Omega_c, \theta)$ is the energy of the polaritonic state (eigenvalue of the QED Hamiltonian). $\boldsymbol{e}$ represents the polarization of the probe field, and may be chosen arbitrarily. This function depends directly on ω, and indirectly on Ω_c and θ (the eigenvalues and eigenvector change depending on these two parameters). The transition matrix element $\mathcal{M}_{I,0}$ represents the matter response to a probe field.

For spatially unstructured cavities, where we use a single effective mode description at $\bar{\boldsymbol{q}} = \boldsymbol{0}$, the transition operator $\hat{P}$ can be written as:

$$\hat{P} = \tilde{A}_{0,\boldsymbol{0}} \sum_{ll',ij} \sum_{\boldsymbol{Q},\nu,\lambda} \mathcal{I}^{\nu,\lambda}_{ll',ij,\boldsymbol{Q},\boldsymbol{0}} \hat{X}^{\nu\dagger}_{ll',ij,\boldsymbol{0}} + h.c.$$

where $\mathcal{I}^{\nu,\lambda}_{ll',ij,\boldsymbol{Q},\boldsymbol{0}}$ is defined in Eq. 28. This quantity was used for Fig. 2a and Fig. 3 of the main text.

For spatially structured cavities, where we use a multi effective mode description of the cavity modes, the the transition operator $\hat{P}$ can be written as:

$$\hat{P} = \sum_{\lambda,\bar{\boldsymbol{q}}} \tilde{A}_{0,\bar{\boldsymbol{q}}} \sum_{ll'} \sum_{i\in C, j\in V} \sum_{\boldsymbol{Q},\nu} \left[\mathcal{I}^{\nu,\lambda}_{ll',ij,\boldsymbol{Q},\bar{\boldsymbol{q}}} \hat{X}^{\nu\dagger}_{ll',ij,\bar{\boldsymbol{q}}} + \mathcal{B}^{\nu,\lambda}_{ll',ij,\boldsymbol{Q},\bar{\boldsymbol{q}}} \hat{X}^{\nu\dagger}_{ll',ij,\boldsymbol{Q}+\bar{\boldsymbol{q}}} \hat{X}^{\nu}_{ll',ij,\boldsymbol{Q}} + h.c. \right]$$

where $\mathcal{B}^{\nu,\lambda}_{ll',ij,\boldsymbol{Q},\bar{\boldsymbol{q}}}$ is defined in Eq. 27. This quantity was used for Fig. 5 of the main text.

B. Spectral function

We define the spectral function $\mathcal{S}(\omega, \Omega_c, \theta, \boldsymbol{Q})$ as:

$$\mathcal{S}(\omega, \Omega_c, \theta, \boldsymbol{Q}) = \sum_I \frac{\langle \Psi_I | \hat{S}^\dagger_{\boldsymbol{Q}} | \Psi_0 \rangle \langle \Psi_0 | \hat{S}_{\boldsymbol{Q}} | \Psi_I \rangle}{\omega - (E_I(\Omega_c, \theta) - E_0(\Omega_c, \theta)) + i\eta} \tag{33}$$

where all quantities and indexes have the same definition as in Section II A. We use the spectral function to obtain the band structure of the polaritonic system in the Moiré BZ. Its physical meaning varies according to the definition of the operator $\hat{S}_{\boldsymbol{Q}}$. If $\hat{S}^\dagger_{\boldsymbol{Q}} = \hat{X}^\dagger_{\boldsymbol{Q}} \otimes \mathbb{I}$, where $\mathbb{I}$ is the identity operator for the photon part, then the spectral function represents the probability of creating an exciton at the *k-point* $\boldsymbol{Q}$ (starting from the polaritonic ground state). This operator is able to capture the interesting features of a Moiré system in the case of a spatially unstructured cavity (i.e. where the photon momentum is zero). This formulation was used for Fig. 2(b-d) and Fig. 4 of the main text.

When plotting the spectral function, we choose a specific path in the Moiré BZ. We use the standard $M - \Gamma - K$ path, which we shorten to $m - \Gamma - \kappa$, where m, κ are taken at the value of the photon momentum along the aforementioned path. Such path is shown in Fig. 1

III. INTERACTION HAMILTONIAN FOR CLASSICALLY DRIVEN CAVITIES

In this section we provide the full expression for the Interaction Hamiltonian (Eq. 6 of the main text) when we model the electromagnetic field inside a cavity as a time-dependent coherent state of light. As in the main text, we will consider only two effective photonic modes in a spatially structured cavity, with momentum $\boldsymbol{q} = [q_x, 0]$, $-\boldsymbol{q} = [-q_x, 0]$. We write the photonic space as:

$$\begin{cases} \left|\tilde{\lambda}_{\boldsymbol{q}}, \tilde{\lambda}_{-\boldsymbol{q}}, t\right\rangle = e^{-\frac{i\left(\omega_{\boldsymbol{q}}+\omega_{-\boldsymbol{q}}\right)t}{2}} \left|e^{-i\omega_{\boldsymbol{q}}t}\tilde{\lambda}_{\boldsymbol{q}}, e^{-i\omega_{-\boldsymbol{q}}t}\tilde{\lambda}_{-\boldsymbol{q}}\right\rangle \\ \hat{a}_{\boldsymbol{q}}\left|\tilde{\lambda}_{\boldsymbol{q}}, \tilde{\lambda}_{-\boldsymbol{q}}, t\right\rangle = \tilde{\lambda}_{\boldsymbol{q}} e^{-\frac{i\left(3\omega_{\boldsymbol{q}}+\omega_{-\boldsymbol{q}}\right)t}{2}} \left|\tilde{\lambda}_{\boldsymbol{q}}, e^{-i\omega_{-\boldsymbol{q}}t}\tilde{\lambda}_{-\boldsymbol{q}}\right\rangle \end{cases}$$

where $\left|\tilde{\lambda}_{\boldsymbol{q}}, \tilde{\lambda}_{-\boldsymbol{q}}, t\right\rangle = \left|\tilde{\lambda}_{\boldsymbol{q}}, t\right\rangle \otimes \left|\tilde{\lambda}_{-\boldsymbol{q}}, t\right\rangle$ and $\left|\tilde{\lambda}_{\boldsymbol{q}}\right\rangle = e^{-\frac{|\tilde{\lambda}_{\boldsymbol{q}}|^2}{2}} \sum_{s=0}^{\infty} \frac{\tilde{\lambda}^s}{\sqrt{s!}} |s_{\boldsymbol{q}}\rangle$. Projecting the interaction Hamiltonian onto such a coherent state leads to:

$$\begin{aligned} \left\langle\tilde{\lambda}_{\boldsymbol{q}}, \tilde{\lambda}_{-\boldsymbol{q}}, t\right| \hat{H}_{\text{int}} \left|\tilde{\lambda}_{\boldsymbol{q}}, \tilde{\lambda}_{-\boldsymbol{q}}, t\right\rangle = \sum_{ll'} \sum_{i\in C, j\in V} \sum_{\boldsymbol{Q},\nu} \sum_{\lambda} \Bigg[\mathcal{M}^{\nu}_{ll',ij,\boldsymbol{Q},\boldsymbol{q}} \hat{X}^{\nu\dagger}_{ll',ij,\boldsymbol{Q}+\boldsymbol{q}} \hat{X}^{\nu}_{ll',ij,\boldsymbol{Q}} + \\ \tilde{A}_{0,\boldsymbol{q}} \left(\mathcal{B}^{\nu,\lambda}_{ll',ij,\boldsymbol{Q},\boldsymbol{q}} \hat{X}^{\nu\dagger}_{ll',ij,\boldsymbol{Q}+\boldsymbol{q}} \hat{X}^{\nu}_{ll',ij,\boldsymbol{Q}} \tilde{\lambda}^*_{\boldsymbol{q}} + \mathcal{B}^{\nu,\lambda}_{ll',ij,\boldsymbol{Q},-\boldsymbol{q}} \hat{X}^{\nu\dagger}_{ll',ij,\boldsymbol{Q}-\boldsymbol{q}} \hat{X}^{\nu}_{ll',ij,\boldsymbol{Q}} \tilde{\lambda}^*_{-\boldsymbol{q}}\right) e^{i\omega_{\boldsymbol{q}}t} + \\ \tilde{A}_{0,\boldsymbol{q}} \left(\mathcal{B}^{\nu,\lambda}_{ll',ij,\boldsymbol{Q},-\boldsymbol{q}} \hat{X}^{\nu\dagger}_{ll',ij,\boldsymbol{Q}-\boldsymbol{q}} \hat{X}^{\nu}_{ll',ij,\boldsymbol{Q}} \tilde{\lambda}_{\boldsymbol{q}} + \mathcal{B}^{\nu,\lambda}_{ll',ij,\boldsymbol{Q},\boldsymbol{q}} \hat{X}^{\nu\dagger}_{ll',ij,\boldsymbol{Q}+\boldsymbol{q}} \hat{X}^{\nu}_{ll',ij,\boldsymbol{Q}} \tilde{\lambda}_{-\boldsymbol{q}}\right) e^{-i\omega_{\boldsymbol{q}}t} + \\ \tilde{A}_{0,\boldsymbol{q}} \left(\mathcal{I}^{\nu,\lambda}_{ll',ij,\boldsymbol{Q},\boldsymbol{q}} \hat{X}^{\nu\dagger}_{ll',ij,\boldsymbol{q}} \tilde{\lambda}^*_{\boldsymbol{q}} + \mathcal{I}^{\nu,\lambda}_{ll',ij,\boldsymbol{Q},-\boldsymbol{q}} \hat{X}^{\nu\dagger}_{ll',ij,-\boldsymbol{q}} \tilde{\lambda}^*_{-\boldsymbol{q}} + h.c.\right) e^{i\omega_{\boldsymbol{q}}t} + \\ \tilde{A}_{0,\boldsymbol{q}} \left(\mathcal{I}^{\nu,\lambda}_{ll',ij,\boldsymbol{Q},-\boldsymbol{q}} \hat{X}^{\nu\dagger}_{ll',ij,-\boldsymbol{q}} \tilde{\lambda}_{\boldsymbol{q}} + \mathcal{I}^{\nu,\lambda}_{ll',ij,\boldsymbol{Q},\boldsymbol{q}} \hat{X}^{\nu\dagger}_{ll',ij,\boldsymbol{q}} \tilde{\lambda}_{-\boldsymbol{q}} + h.c.\right) e^{-i\omega_{\boldsymbol{q}}t} \Bigg] \end{aligned} \tag{34}$$

A. High frequency limit

In this section, we discuss the high frequency limit for the classically driven Hamiltonian. In the following, we consider a classically driven cavity with $\tilde{A}_0 = 0.02 a.u.$ and two modes with momentum $\boldsymbol{q} = [0.009, 0]$, $-\boldsymbol{q} = [-0.009, 0]$. Since we are interested in studying the effect of the classical driving, we set the Moiré potential $\mathcal{M}^{\nu}_{ll',ij,\boldsymbol{Q}\boldsymbol{q}} = 0$.

In the main text, we state that in the high frequency regime the external driving cannot modify the parabolic dispersion. In fact, the effective Floquet Hamiltonian is given by the Van Vleck expansion [8]:

$$H_{\text{eff}} = H^{(n=0)} + \frac{\left[H^{(n=-1)}, H^{(n=1)}\right]}{\omega} + \mathcal{O}\left(\frac{1}{\omega^2}\right) \tag{35}$$

where n is the Floquet frequency index. Clearly, if $\omega \to \infty$ one has that $H_{\text{eff}} = H^{(n=0)}$. We show such progression in Fig. 2, where we plot the spectral function for a for various values of the modes frequency. At small values of ω, the positive and negative frequencies replicas from the Floquet Hamiltonian mix with the excitonic dispersion, generating a rich spectrum. As ω increases, the spectral function pictures a parabolic dispersion, meaning that the exciton is unperturbed.

In the following, we provide the complete expression for the commutator $\left[H^{(n=-1)}, H^{(n=1)}\right]$. $H^{(n=1)}$ ($H^{(n=-1)}$) contains all terms from Eq. 34 that are associated to $e^{i\omega_{\boldsymbol{q}}t}$ ($e^{-i\omega_{\boldsymbol{q}}t}$). Note that since we are interested in studying the terms that originate the Moiré-like confinement, we will focus only on the conserving term of the bilinear coupling $\mathcal{B}^{\nu,\lambda}_{ll',ij,\boldsymbol{Q},\boldsymbol{q}}$. This is justified because the conserving term $\mathcal{I}^{\nu,\lambda}_{ll',ij,\boldsymbol{Q},\boldsymbol{q}}$ couples the ground state, and it signature on the spectrum is the Rabi splitting. Hence:

$$\begin{aligned} \left[H^{(n=-1)}, H^{(n=1)}\right] = \sum_{ll'} \sum_{i\in C, j\in V} \sum_{\boldsymbol{Q},\nu,\lambda} \tilde{A}^2_{0,\boldsymbol{q}} \Bigg[\\ \left(\mathcal{B}^{\nu,\lambda}_{ll',ij,\boldsymbol{Q},-\boldsymbol{q}} \mathcal{B}^{\nu,\lambda}_{ll',ij,\boldsymbol{Q},\boldsymbol{q}} |\tilde{\lambda}_{\boldsymbol{q}}|^2 - \mathcal{B}^{\nu,\lambda}_{ll',ij,\boldsymbol{Q},-\boldsymbol{q}} \mathcal{B}^{\nu,\lambda}_{ll',ij,\boldsymbol{Q},\boldsymbol{q}} |\tilde{\lambda}_{-\boldsymbol{q}}|^2\right) \hat{X}^{\nu\dagger}_{ll',ij,\boldsymbol{Q}-\boldsymbol{q}} \hat{X}^{\nu}_{ll',ij,\boldsymbol{Q}} \hat{X}^{\nu\dagger}_{ll',ij,\boldsymbol{Q}+\boldsymbol{q}} \hat{X}^{\nu}_{ll',ij,\boldsymbol{Q}} + \\ \left(\mathcal{B}^{\nu,\lambda}_{ll',ij,\boldsymbol{Q},-\boldsymbol{q}} \mathcal{B}^{\nu,\lambda}_{ll',ij,\boldsymbol{Q},\boldsymbol{q}} |\tilde{\lambda}_{-\boldsymbol{q}}|^2 - \mathcal{B}^{\nu,\lambda}_{ll',ij,\boldsymbol{Q},-\boldsymbol{q}} \mathcal{B}^{\nu,\lambda}_{ll',ij,\boldsymbol{Q},\boldsymbol{q}} |\tilde{\lambda}_{\boldsymbol{q}}|^2\right) \hat{X}^{\nu\dagger}_{ll',ij,\boldsymbol{Q}+\boldsymbol{q}} \hat{X}^{\nu}_{ll',ij,\boldsymbol{Q}} \hat{X}^{\nu\dagger}_{ll',ij,\boldsymbol{Q}-\boldsymbol{q}} \hat{X}^{\nu}_{ll',ij,\boldsymbol{Q}} \Bigg] \end{aligned}$$

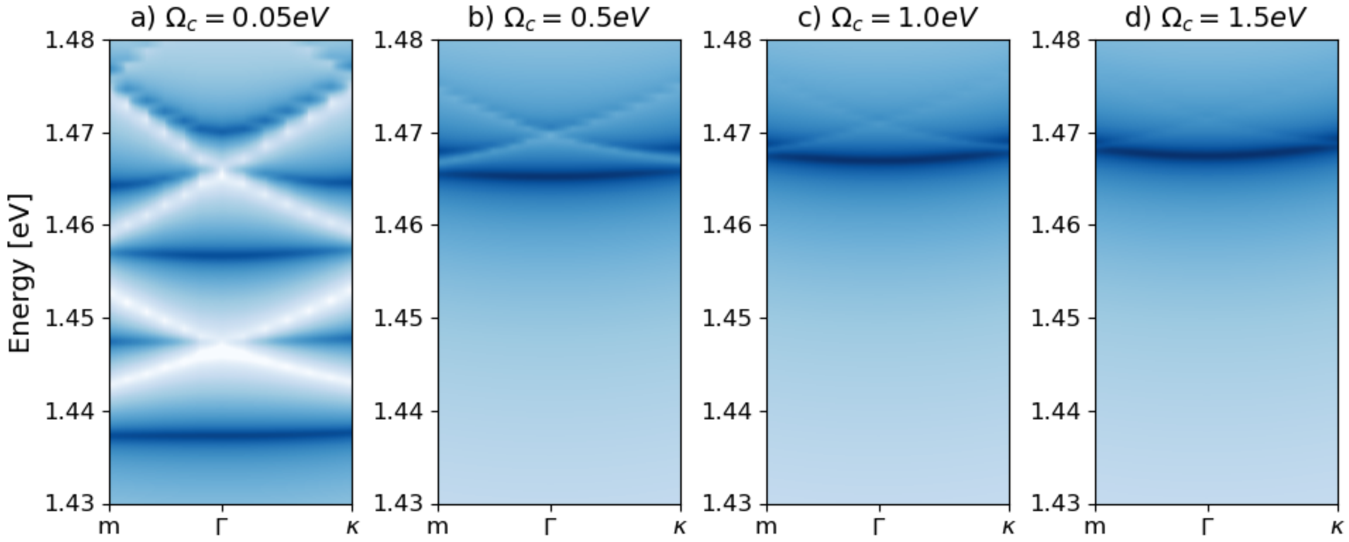

SUPPLEMENTARY FIGURE 2. **Effective Floquet Hamiltonian for different cavity modes frequencies**. Spectral function for classically driven cavity with two modes, for various values of the modes' energy. In the high frequency regime, the effect of the effective Floquet Hamiltonian vanishes, as predicted by the Van Vleck expansion (Eq. 35). (**a**) Low frequency regime, where the effective Floquet Hamiltonian dominates the spectrum. (**b, c**) As the frequency of the cavity modes increases, the effective Floquet Hamiltonian vanishes, decaying as $\frac{1}{\omega}$. (**d**) In the last panel of the series, the effective Floquet Hamiltonian is almost negligible, and only a parabolic band corresponding to $H^{(n=0)}$ remains.

which is non-zero only when $|\tilde{\lambda}_{\boldsymbol{q}}|^2 \neq |\tilde{\lambda}_{-\boldsymbol{q}}|^2$. Since for a coherent state of light one typically has that $|\tilde{\lambda}_{\boldsymbol{q}}|^2 \approx |\tilde{\lambda}_{-\boldsymbol{q}}|^2$, to observe a first order correction ω must be small.

IV. HAMILTONIAN DOWN-FOLDING

This appendix discusses the downfolding of the QED Hamiltonian in a dressed photon space. This approach allows us to define an effective Hamiltonian, composed of a single photon sector, that contains the action of the full QED Hamiltonian [9]. The first-order expansion of such an approximation for a system with N modes can be written as [6]:

$$\begin{aligned} \hat{H}_{\text{eff}} = & \langle 0_1, \cdots, 0_N | \hat{H} | 0_1, \cdots, 0_N \rangle - \\ & \sum_{\alpha}^{N} \frac{1}{\omega_\alpha} \Big[\langle 0_1, \cdots, 0_N | \hat{H} | 0_1, \cdots, 1_\alpha, \cdots, 0_N \rangle \cdot \langle 0_1, \cdots, 1_\alpha, \cdots, 0_N | \hat{H} | 0_1, \cdots, 0_N \rangle \Big] + \mathcal{O}\left(\frac{1}{\omega^2}\right) \end{aligned} \tag{36}$$

where $\hat{H}$ is the Hamiltonian one wants to approximate.

In the following we apply this expansion to the QED Hamiltonian (defined in the main text), for a system with two modes $\boldsymbol{q}$ and $-\boldsymbol{q}$. We will not focus on the free exciton Hamiltonian nor on the uncoupled photon Hamiltonian because we are interested on studying how the interacting terms are transformed by the down-folding.

Let us first consider the zeroth-order correction. The terms associated with the bilinear coupling have a photonic creation or annihilation operator, thus will not give any contribution. Hence:

$$\langle 0_{\boldsymbol{q}}, 0_{-\boldsymbol{q}} | \hat{H}_m | 0_{\boldsymbol{q}}, 0_{-\boldsymbol{q}} \rangle = \sum_{ll'} \sum_{i \in C, j \in V} \sum_{\boldsymbol{Q}, \boldsymbol{q}, \nu} \mathcal{M}^{\nu}_{ll', ij, \boldsymbol{Q}, \boldsymbol{q}} \hat{X}^{\nu \dagger}_{ll', ij, \boldsymbol{Q}+\boldsymbol{q}} \hat{X}^{\nu}_{ll', ij, \boldsymbol{Q}} \tag{37}$$

where $\hat{H}_m$ is the Moiré term in the QED Hamiltonian.

Let us now focus on the first-order correction. This time, the term associated with the Moiré potential will not give any contribution. Therefore, only the bilinear coupling terms will contribute (both the conserving, associated with the coefficient $\mathcal{B}$ and the non-conserving one, associated with the coefficient $\mathcal{I}$).

First, we consider the conserving term:

$$\hat{H}_{bil,c} = \sum_{\lambda, \bar{\boldsymbol{q}}} \tilde{A}_{0, \bar{\boldsymbol{q}}} \sum_{ll'} \sum_{i \in C, j \in V} \sum_{\boldsymbol{Q}, \nu} \mathcal{B}^{\nu, \lambda}_{ll', ij, \boldsymbol{Q}, \bar{\boldsymbol{q}}} \hat{X}^{\nu \dagger}_{ll', ij, \boldsymbol{Q}+\bar{\boldsymbol{q}}} \hat{X}^{\nu}_{ll', ij, \boldsymbol{Q}} \left(\hat{a}^{\dagger}_{\bar{\boldsymbol{q}}, \lambda} + \hat{a}_{-\bar{\boldsymbol{q}}, \lambda} \right)$$

Since $\hat{H}_{bil,c}$ contains a photonic creation and annihilation operator, the zeroth-order expansion yields $\langle 0_{\boldsymbol{q}}, 0_{-\boldsymbol{q}}| \hat{H}_{bil,c} |0_{\boldsymbol{q}}, 0_{-\boldsymbol{q}}\rangle = 0$. The first-order correction reads:

$$\langle 0_{\boldsymbol{q}}, 0_{-\boldsymbol{q}}| \hat{H}_{bil,c} |1_{\boldsymbol{q}}, 0_{-\boldsymbol{q}}\rangle = \tilde{A}_{0,-\boldsymbol{q}} \sum_{ll'} \sum_{i\in C, j\in V} \sum_{\boldsymbol{Q},\nu} \sum_{\lambda} \mathcal{B}^{\nu,\lambda}_{ll',ij,\boldsymbol{Q},-\boldsymbol{q}} \hat{X}^{\nu\dagger}_{ll',ij,\boldsymbol{Q}-\boldsymbol{q}} \hat{X}^{\nu}_{ll',ij,\boldsymbol{Q}} \tag{38}$$

$$\langle 1_{\boldsymbol{q}}, 0_{-\boldsymbol{q}}| \hat{H}_{bil,c} |0_{\boldsymbol{q}}, 0_{-\boldsymbol{q}}\rangle = \tilde{A}_{0,\boldsymbol{q}} \sum_{ll'} \sum_{i\in C, j\in V} \sum_{\boldsymbol{Q},\nu} \sum_{\lambda} \mathcal{B}^{\nu,\lambda}_{ll',ij,\boldsymbol{Q},\boldsymbol{q}} \hat{X}^{\nu\dagger}_{ll',ij,\boldsymbol{Q}+\boldsymbol{q}} \hat{X}^{\nu}_{ll',ij,\boldsymbol{Q}} \tag{39}$$

So:

$$\begin{aligned} \frac{\hat{H}^{bil,c}_{10,\boldsymbol{q}} \hat{H}^{bil,c,\dagger}_{10,\boldsymbol{q}}}{\omega_{\boldsymbol{q}}} &= \frac{\langle 0_{\boldsymbol{q}}, 0_{-\boldsymbol{q}}| \hat{H}_{bil,c} |1_{\boldsymbol{q}}, 0_{-\boldsymbol{q}}\rangle \cdot \langle 1_{\boldsymbol{q}}, 0_{-\boldsymbol{q}}| \hat{H}_{bil,c} |0_{\boldsymbol{q}}, 0_{-\boldsymbol{q}}\rangle}{\omega_{\boldsymbol{q}}} = \\ \frac{\tilde{A}_{0,\boldsymbol{q}} \tilde{A}_{0,-\boldsymbol{q}}}{\omega_{\boldsymbol{q}}} &\sum_{ll', l_1 l_1'} \sum_{ij, i_1 j_1} \sum_{\boldsymbol{Q}, \boldsymbol{Q_1}, \nu, \nu_1} \mathcal{B}^{\nu,\lambda}_{ll',ij,\boldsymbol{Q},-\boldsymbol{q}} \mathcal{B}^{\nu_1,\lambda}_{l_1 l_1',i_1 j_1,\boldsymbol{Q_1},\boldsymbol{q}} \hat{X}^{\nu\dagger}_{ll',ij,\boldsymbol{Q}-\boldsymbol{q}} \hat{X}^{\nu_1\dagger}_{l_1 l_1',i_1 j_1,\boldsymbol{Q_1}+\boldsymbol{q}} \hat{X}^{\nu}_{ll',ij,\boldsymbol{Q}} \hat{X}^{\nu_1}_{l_1 l_1',i_1 j_1,\boldsymbol{Q_1}} \end{aligned} \tag{40}$$

Equivalently, since $\omega_{\boldsymbol{q}} = \omega_{-\boldsymbol{q}}$ computing

$$\frac{\hat{H}^{bil,c}_{10,-\boldsymbol{q}} \hat{H}^{bil,c,\dagger}_{10,-\boldsymbol{q}}}{\omega_{-\boldsymbol{q}}} = \frac{\langle 0_{\boldsymbol{q}}, 0_{-\boldsymbol{q}}| \hat{H}_{bil,c} |0_{\boldsymbol{q}}, 1_{-\boldsymbol{q}}\rangle \cdot \langle 0_{\boldsymbol{q}}, 1_{-\boldsymbol{q}}| \hat{H}_{bil,c} |0_{\boldsymbol{q}}, 0_{-\boldsymbol{q}}\rangle}{\omega_{-\boldsymbol{q}}}$$

leads to the same result. Hence, the full first-order correction for the conserving term reads:

$$\begin{aligned} &\frac{\hat{H}^{bil,c}_{10,\boldsymbol{q}} \hat{H}^{bil,c,\dagger}_{10,\boldsymbol{q}}}{\omega_{\boldsymbol{q}}} + \frac{\hat{H}^{bil,c}_{10,-\boldsymbol{q}} \hat{H}^{bil,c,\dagger}_{10,-\boldsymbol{q}}}{\omega_{-\boldsymbol{q}}} = \\ &\frac{2\tilde{A}_{0,\boldsymbol{q}} \tilde{A}_{0,-\boldsymbol{q}}}{\omega_{\boldsymbol{q}}} \sum_{ll', l_1 l_1'} \sum_{ij, i_1 j_1} \sum_{\boldsymbol{Q}, \boldsymbol{Q_1}, \nu, \nu_1} \mathcal{B}^{\nu,\lambda}_{ll',ij,\boldsymbol{Q},-\boldsymbol{q}} \mathcal{B}^{\nu_1,\lambda}_{l_1 l_1',i_1 j_1,\boldsymbol{Q_1},\boldsymbol{q}} \hat{X}^{\nu\dagger}_{ll',ij,\boldsymbol{Q}-\boldsymbol{q}} \hat{X}^{\nu_1\dagger}_{l_1 l_1',i_1 j_1,\boldsymbol{Q_1}+\boldsymbol{q}} \hat{X}^{\nu}_{ll',ij,\boldsymbol{Q}} \hat{X}^{\nu_1}_{l_1 l_1',i_1 j_1,\boldsymbol{Q_1}} \end{aligned} \tag{41}$$

Let us now expand the non-conserving term of the bi-linear coupling:

$$\hat{H}_{bil,nc} = \sum_{\lambda,\bar{\boldsymbol{q}}} \tilde{A}_{0,\bar{\boldsymbol{q}}} \sum_{ll'} \sum_{i\in C, j\in V} \sum_{\boldsymbol{Q},\nu} \mathcal{I}^{\nu,\lambda}_{ll',ij,\boldsymbol{Q},\bar{\boldsymbol{q}}} \hat{X}^{\nu\dagger}_{ll',ij,\bar{\boldsymbol{q}}} \left(\hat{a}^{\dagger}_{\bar{\boldsymbol{q}},\lambda} + \hat{a}_{-\bar{\boldsymbol{q}},\lambda}\right) + h.c.$$

Since $\hat{H}_{bil,nc}$ contains a photonic creation and annihilation operator, the zeroth-order expansion yields $\langle 0_{\boldsymbol{q}}, 0_{-\boldsymbol{q}}| \hat{H}_{bil,nc} |0_{\boldsymbol{q}}, 0_{-\boldsymbol{q}}\rangle = 0$. Conversely, the first-order correction reads:

$$\langle 0_{\boldsymbol{q}}, 0_{-\boldsymbol{q}}| \hat{H}_{bil,nc} |1_{\boldsymbol{q}}, 0_{-\boldsymbol{q}}\rangle = \tilde{A}_{0,-\boldsymbol{q}} \sum_{ll'} \sum_{i\in C, j\in V} \sum_{\boldsymbol{Q},\nu} \sum_{\lambda} \mathcal{I}^{\nu,\lambda}_{ll',ij,\boldsymbol{Q},-\boldsymbol{q}} \hat{X}^{\nu\dagger}_{ll',ij,-\boldsymbol{q}} + \tilde{A}_{0,\boldsymbol{q}} \sum_{ll'} \sum_{i\in C, j\in V} \sum_{\boldsymbol{Q},\nu} \sum_{\lambda} \mathcal{I}^{\nu,\lambda}_{ll',ij,\boldsymbol{Q},\boldsymbol{q}} \hat{X}^{\nu}_{ll',ij,\boldsymbol{q}}$$

$$\langle 1_{\boldsymbol{q}}, 0_{-\boldsymbol{q}}| \hat{H}_{bil,nc} |0_{\boldsymbol{q}}, 0_{-\boldsymbol{q}}\rangle = \tilde{A}_{0,\boldsymbol{q}} \sum_{ll'} \sum_{i\in C, j\in V} \sum_{\boldsymbol{Q},\nu} \sum_{\lambda} \mathcal{I}^{\nu,\lambda}_{ll',ij,\boldsymbol{Q},\boldsymbol{q}} \hat{X}^{\nu\dagger}_{ll',ij,\boldsymbol{q}} + \tilde{A}_{0,-\boldsymbol{q}} \sum_{ll'} \sum_{i\in C, j\in V} \sum_{\boldsymbol{Q},\nu} \sum_{\lambda} \mathcal{I}^{\nu,\lambda}_{ll',ij,\boldsymbol{Q},-\boldsymbol{q}} \hat{X}^{\nu}_{ll',ij,-\boldsymbol{q}}$$

So:

$$\begin{aligned} \frac{\hat{H}^{bil,nc}_{10,\boldsymbol{q}} \hat{H}^{bil,nc,\dagger}_{10,\boldsymbol{q}}}{\omega_{\boldsymbol{q}}} &= \frac{\langle 0_{\boldsymbol{q}}, 0_{-\boldsymbol{q}}| \hat{H}_{bil,nc} |1_{\boldsymbol{q}}, 0_{-\boldsymbol{q}}\rangle \cdot \langle 1_{\boldsymbol{q}}, 0_{-\boldsymbol{q}}| \hat{H}_{bil,nc} |0_{\boldsymbol{q}}, 0_{-\boldsymbol{q}}\rangle}{\omega_{\boldsymbol{q}}} = \\ \frac{\tilde{A}_{0,\boldsymbol{q}} \tilde{A}_{0,-\boldsymbol{q}}}{\omega_{\boldsymbol{q}}} &\sum_{ll', l_1 l_1'} \sum_{ij, i_1 j_1} \sum_{\boldsymbol{Q}, \boldsymbol{Q_1}, \nu, \nu_1} \mathcal{I}^{\nu,\lambda}_{ll',ij,\boldsymbol{Q},\boldsymbol{q}} \mathcal{I}^{\nu_1,\lambda,*}_{l_1 l_1',i_1 j_1,\boldsymbol{Q_1},\boldsymbol{q}} \hat{X}^{\nu\dagger}_{ll',ij,\boldsymbol{q}} \hat{X}^{\nu_1}_{l_1 l_1',i_1 j_1,\boldsymbol{q}} + \\ \frac{\tilde{A}_{0,\boldsymbol{q}} \tilde{A}_{0,-\boldsymbol{q}}}{\omega_{\boldsymbol{q}}} &\sum_{ll', l_1 l_1'} \sum_{ij, i_1 j_1} \sum_{\boldsymbol{Q}, \boldsymbol{Q_1}, \nu, \nu_1} \mathcal{I}^{\nu,\lambda,*}_{ll',ij,\boldsymbol{Q},-\boldsymbol{q}} \mathcal{I}^{\nu_1,\lambda}_{l_1 l_1',i_1 j_1,\boldsymbol{Q_1},-\boldsymbol{q}} \hat{X}^{\nu}_{ll',ij,-\boldsymbol{q}} \hat{X}^{\nu_1\dagger}_{l_1 l_1',i_1 j_1,-\boldsymbol{q}} \end{aligned} \tag{42}$$

Value	Sub1	$MoSe_2$	Interlayer	WSe_2	Sub2
Thickness [A]		6.2926	6.22705	6.1615	
Lattice param [A]		3.32		3.319	
Dielectric const	4	16.5	1	15.1	4
Hole eff mass		0.6		0.36	
Elec eff mass		0.5		0.29	

SUPPLEMENTARY TABLE I. The numerical values (in atomic units) for the parameters required to compute the Coulomb potential.

where we disregarded the terms involving a double creation or annihilation excitonic operators. This can be rewritten as:

$$\frac{\hat{H}^{bil,nc}_{10,\boldsymbol{q}}\hat{H}^{bil,nc,\dagger}_{10,\boldsymbol{q}}}{\omega_{\boldsymbol{q}}} = \frac{\langle 0_{\boldsymbol{q}}, 0_{-\boldsymbol{q}}| \hat{H}_{bil,nc} |1_{\boldsymbol{q}}, 0_{-\boldsymbol{q}}\rangle \cdot \langle 1_{\boldsymbol{q}}, 0_{-\boldsymbol{q}}| \hat{H}_{bil,nc} |0_{\boldsymbol{q}}, 0_{-\boldsymbol{q}}\rangle}{\omega_{\boldsymbol{q}}} = \frac{\tilde{A}_{0,\boldsymbol{q}}\tilde{A}_{0,-\boldsymbol{q}}}{\omega_{\boldsymbol{q}}} \sum_{ll',l_1l'_1} \sum_{ij,i_1j_1} \sum_{\boldsymbol{q},\boldsymbol{Q},\boldsymbol{Q}_1,\nu,\nu_1} \mathcal{I}^{\nu,\lambda}_{ll',ij,\boldsymbol{Q},\boldsymbol{q}} \mathcal{I}^{\nu_1,\lambda,*}_{l_1l'_1,i_1j_1,\boldsymbol{Q_1},\boldsymbol{q}} \hat{X}^{\nu\dagger}_{ll',ij,\boldsymbol{q}} \hat{X}^{\nu_1}_{l_1l'_1,i_1j_1,\boldsymbol{q}} \tag{43}$$

Equivalently, since $\omega_{\boldsymbol{q}} = \omega_{-\boldsymbol{q}}$ computing

$$\frac{\hat{H}^{bil,nc}_{10,-\boldsymbol{q}}\hat{H}^{bil,nc,\dagger}_{10,-\boldsymbol{q}}}{\omega_{-\boldsymbol{q}}} = \frac{\langle 0_{\boldsymbol{q}}, 0_{-\boldsymbol{q}}| \hat{H}_{bil,nc} |0_{\boldsymbol{q}}, 1_{-\boldsymbol{q}}\rangle \cdot \langle 0_{\boldsymbol{q}}, 1_{-\boldsymbol{q}}| \hat{H}_{bil,nc} |0_{\boldsymbol{q}}, 0_{-\boldsymbol{q}}\rangle}{\omega_{-\boldsymbol{q}}}$$

leads to the same result. Hence, the full first-order correction for the non-conserving term reads:

$$\frac{\hat{H}^{bil,nc}_{10,\boldsymbol{q}}\hat{H}^{bil,nc,\dagger}_{10,\boldsymbol{q}}}{\omega_{\boldsymbol{q}}} + \frac{\hat{H}^{bil,nc}_{10,-\boldsymbol{q}}\hat{H}^{bil,nc,\dagger}_{10,-\boldsymbol{q}}}{\omega_{-\boldsymbol{q}}} = \frac{2\tilde{A}_{0,\boldsymbol{q}}\tilde{A}_{0,-\boldsymbol{q}}}{\omega_{\boldsymbol{q}}} \sum_{ll',l_1l'_1} \sum_{ij,i_1j_1} \sum_{\boldsymbol{q},\boldsymbol{Q},\boldsymbol{Q}_1,\nu,\nu_1} \mathcal{I}^{\nu,\lambda}_{ll',ij,\boldsymbol{Q},\boldsymbol{q}} \mathcal{I}^{\nu_1,\lambda,*}_{l_1l'_1,i_1j_1,\boldsymbol{Q_1},\boldsymbol{q}} \hat{X}^{\nu\dagger}_{ll',ij,\boldsymbol{q}} \hat{X}^{\nu_1}_{l_1l'_1,i_1j_1,\boldsymbol{q}} \tag{44}$$

Finally, the full down-folded Hamiltonian can be obtained by summing Eq. 37, 41 and 44.

V. METHODS AND COMPUTATIONAL DETAILS

A. Mott-Wannier model computational details

Excitons in the Mott-Wannier model are formed thanks to the solution of Eq. 16:

$$\frac{\hbar^2 k^2}{2m^r_{ll'}} \psi^{\nu}_{ll'}(\boldsymbol{k}) - \sum_{\boldsymbol{q}} \mathcal{W}^{ll'}_{\boldsymbol{q}} \psi^{\nu}_{ll'}(\boldsymbol{k}+\boldsymbol{q}) = \hat{E}^{\nu}_{ll'} \psi^{\nu}_{ll'}(\boldsymbol{k})$$

where $\mathcal{W}^{ll'}_{\boldsymbol{q}}$ is the Coulomb potential defined in 1 and $\hat{E}^{\nu}_{ll'}$ is the bound energy. To solve this equation, we model the Coulomb potential after Eq. 2 of the Supplementary Information of Ref. [3]. Table I reports the numerical values used. We build a 65×65 k-points grid around Γ and after solving equation we obtain the excitonic wavefunctions $\psi^{\nu}_{ll'}(\boldsymbol{k})$ and the bound energies $\hat{E}^{\nu}_{ll'}$.

The use of unmodified Mott-Wannier exciton states assumes that the mixing between the $\nu = 1s$ excitonic state and higher excited states is small. This approximation is justified as long as the the ratio between the interaction energy and the energy difference between such states is significantly less than 1. Note that including the excitonic $2s$ implies a major increase in the computational cost.

Moreover, we assume that the cavity field does not significantly distort the internal exciton wavefunction. Such an approximation is justified as long as long the light matter interaction does not distort the internal degrees of freedom of the exciton (i.e. the relative position of the electron and of the hole). Hence, the spatial variation of the cavity field, which is determined by the periodicity of the grating, must be small relative to the exciton size (i.e. to the excitonic radius), which is the case for the investigated periodicites.

B. QED Hamiltonian approximations

The QED Hamiltonian is reported in Eq. 3 of the main text. We represent this Hamiltonian on the basis $|\Psi_{EX}\rangle \otimes |n\rangle_0 \otimes |n\rangle_1$ ..., where $|\Psi_{EX}\rangle$ is a Slater determinant representing an excitonic excitation or the many-body ground state and $|n\rangle_i$ represents a cavity mode. For each mode, we only consider the vacuum and one-photon state: $\{|0\rangle, |1\rangle\}$. In the numerical simulations of this work, we make approximations to reduce the sums in the QED Hamiltonian. In particular, we consider only the first excitonic state, thus $\nu = 1s$. We also consider only one valence band and one conduction band, thus the indexes i, j can be disregarded. All simulations presented in the paper are done for the intra-layer exciton of the $MoSe_2$ layer, thus also the indexes l, l' can be dropped. Note that we tried to simulate the coupling to all excitons together, and we noticed that the physics of each is not affected by the presence of other excitons (at least to a first order approximation). Despite not including the two layers explicitly, the system can still be considered a bi-layer because the effect of WSe_2 on $MoSe_2$ is included in the excitonic states $\psi(\boldsymbol{k})$ and corresponding eigenenergies and in the Moiré potential $\mathcal{M}_{\boldsymbol{Q},\boldsymbol{q}}$. It is important to note that in principle one should not separate the different types of excitons as they can mix through the interaction with light. However, we verified that for the sake of what presented in this work, that mixing is not relevant. Finally, we absorbed the diamagnetic term into the uncoupled photon Hamiltonian by performing a Bogoliubov transformation [6, 10].
Considering all assumptions above, the Hamiltonian we implemented in our code is the following:

$$\begin{aligned}\hat{H}_{QED} = \sum_{\bar{\boldsymbol{q}},\lambda} \omega_{\bar{\boldsymbol{q}}} \left(\hat{a}^\dagger_{\bar{\boldsymbol{q}},\lambda} \hat{a}_{\bar{\boldsymbol{q}},\lambda} + \frac{1}{2} \right) + \sum_{\boldsymbol{Q}} \mathcal{E}_{\boldsymbol{Q}} \hat{X}^\dagger_{\boldsymbol{Q}} \hat{X}_{\boldsymbol{Q}} + \sum_{\boldsymbol{Q},\boldsymbol{q}} \mathcal{M}_{\boldsymbol{q}} \hat{X}^\dagger_{\boldsymbol{Q}+\boldsymbol{q}} \hat{X}_{\boldsymbol{Q}} + \\ \sum_{\lambda,\bar{\boldsymbol{q}}} \tilde{A}_{0,\bar{\boldsymbol{q}}} \sum_{\boldsymbol{Q}} \mathcal{B}^\lambda_{\boldsymbol{Q},\bar{\boldsymbol{q}}} \hat{X}^\dagger_{\boldsymbol{Q}+\bar{\boldsymbol{q}}} \hat{X}_{\boldsymbol{Q}} \left(\hat{a}^\dagger_{\bar{\boldsymbol{q}},\lambda} + \hat{a}_{-\bar{\boldsymbol{q}},\lambda} \right) + \sum_{\lambda,\bar{\boldsymbol{q}}} \tilde{A}_{0,\bar{\boldsymbol{q}}} \sum_{\boldsymbol{Q}} \mathcal{I}^\lambda_{\boldsymbol{Q},\bar{\boldsymbol{q}}} \hat{X}^\dagger_{\boldsymbol{Q}} \left(\hat{a}^\dagger_{\bar{\boldsymbol{q}},\lambda} + \hat{a}_{-\bar{\boldsymbol{q}},\lambda} \right) + h.c.\end{aligned} \tag{45}$$

We solve this Hamiltonian by building its matrix representation on the aforementioned basis and performing an exact diagonalization, which gives us access to the polaritonic eigenvalues and eigenstates.

C. Transition matrix elements

The transition matrix elements are given by computing the quantity

$$\langle \Psi_{EX,\boldsymbol{Q}}, n_{\bar{\boldsymbol{q}}} | \hat{H}_{bil} | \Psi_{EX,\boldsymbol{q}'}, m_{\bar{\boldsymbol{q}}} \rangle$$

where $|n_{\bar{\boldsymbol{q}}}\rangle, |m_{\bar{\boldsymbol{q}}}\rangle$ represent the initial and final photonic state for the mode $\bar{\boldsymbol{q}}$, and $\hat{H}_{bil}$ is:

$$\hat{H}_{bil} = \sum_{\lambda,\bar{\boldsymbol{q}}} \tilde{A}_{0,\bar{\boldsymbol{q}}} \sum_{\boldsymbol{Q}} \mathcal{B}^\lambda_{\boldsymbol{Q},\bar{\boldsymbol{q}}} \hat{X}^\dagger_{\boldsymbol{Q}+\bar{\boldsymbol{q}}} \hat{X}_{\boldsymbol{Q}} \left(\hat{a}^\dagger_{\bar{\boldsymbol{q}},\lambda} + \hat{a}_{-\bar{\boldsymbol{q}},\lambda} \right) + \sum_{\lambda,\bar{\boldsymbol{q}}} \tilde{A}_{0,\bar{\boldsymbol{q}}} \sum_{\boldsymbol{Q}} \mathcal{I}^\lambda_{\boldsymbol{Q},\bar{\boldsymbol{q}}} \hat{X}^\dagger_{\boldsymbol{Q}} \left(\hat{a}^\dagger_{\bar{\boldsymbol{q}},\lambda} + \hat{a}_{-\bar{\boldsymbol{q}},\lambda} \right) + h.c.$$

As for the photonic part, it is immediate to see that the matrix element is non-zero only if $|n_{\bar{\boldsymbol{q}}}\rangle = |m_{\bar{\boldsymbol{q}}}\rangle \pm 1$. As for the matter part, we shall distinguish between $\bar{\boldsymbol{q}}$ equal to zero (only vertical transitions are allowed) or finite (the photon transfers its momentum to the matter). In the former case, which throughout the paper is referred to as a spatially unstructured cavity, one has that only

$$\mathcal{I}^\lambda_{\boldsymbol{Q},\bar{\boldsymbol{q}}} = \sum_{\boldsymbol{k}} p^\lambda_{\boldsymbol{k}+\bar{\boldsymbol{q}},\boldsymbol{k}} \psi \left[\alpha\left(\boldsymbol{k}\right) + \beta\left(\boldsymbol{k}-\boldsymbol{Q}\right) \right]$$

contributes to the coupling. In TMDs, excitons are very localized in some points of the Brillouin zone, thus one can approximate that the valence-conduction momentum element $p_{\boldsymbol{k}+\bar{\boldsymbol{q}},\boldsymbol{k}}$ is constant with $\boldsymbol{k}$. In particular, we choose its value at K as reference. Thus, we compute $\mathcal{I}^\lambda_{\boldsymbol{Q},\bar{\boldsymbol{q}}} = p^\lambda_{K+\bar{\boldsymbol{q}},K} \sum_{\boldsymbol{k}} \psi \left[\alpha\left(\boldsymbol{k}\right) + \beta\left(\boldsymbol{k}-\boldsymbol{Q}\right) \right]$.
In contrast, if $\bar{\boldsymbol{q}}$ is not zero, which throughout the paper is referred to as a spatially structured cavity, one should also account for

$$\mathcal{B}^\lambda_{\boldsymbol{Q},\bar{\boldsymbol{q}}} = \sum_{\boldsymbol{k}} \left[p^{cc,\lambda}_{\boldsymbol{k}+\bar{\boldsymbol{q}},\boldsymbol{k}} \Psi\left(\boldsymbol{k}-\boldsymbol{Q}, \boldsymbol{k}+\bar{\boldsymbol{q}}, \boldsymbol{k}-\boldsymbol{Q}, \boldsymbol{k}\right) - p^{vv,\lambda}_{\boldsymbol{k}+\bar{\boldsymbol{q}},\boldsymbol{k}} \Psi\left(\boldsymbol{k}, \boldsymbol{k}-\boldsymbol{Q}, \boldsymbol{k}+\bar{\boldsymbol{q}}, \boldsymbol{k}-\boldsymbol{Q}\right) \right]$$

By assuming that the valence and conduction band are parabolic, we can simplify the expression above as $p^{cc,\lambda}_{\boldsymbol{k}+\bar{\boldsymbol{q}},\boldsymbol{k}} = -p^{vv,\lambda}_{\boldsymbol{k}+\bar{\boldsymbol{q}},\boldsymbol{k}} = \frac{\|\bar{\boldsymbol{q}}\|}{m_{\text{eff}}}$ [5], where m_{eff} is the excitonic effective mass. Hence,

$$\mathcal{B}_{\boldsymbol{Q},\bar{\boldsymbol{q}}} = \frac{\|\bar{\boldsymbol{q}}\|}{m_{\text{eff}}} \sum_{\boldsymbol{k}} \left[\Psi\left(\boldsymbol{k}-\boldsymbol{Q}, \boldsymbol{k}+\bar{\boldsymbol{q}}, \boldsymbol{k}-\boldsymbol{Q}, \boldsymbol{k}\right) + \Psi\left(\boldsymbol{k}, \boldsymbol{k}-\boldsymbol{Q}, \boldsymbol{k}+\bar{\boldsymbol{q}}, \boldsymbol{k}-\boldsymbol{Q}\right) \right]$$

[1] S. Brem, C. Linderälv, P. Erhart, and E. Malic, Tunable phases of moiré excitons in van der waals heterostructures, Nano Letters **20**, 8534–8540 (2020).

[2] M. K. Svendsen, M. Ruggenthaler, H. Hübener, C. Schäfer, M. Eckstein, A. Rubio, and S. Latini, Theory of quantum light-matter interaction in cavities: Extended systems and the long wavelength approximation, arXiv https://doi.org/10.48550/arXiv.2312.17374 (2023), arXiv:2312.17374.

[3] S. Ovesen, S. Brem, C. Linderälv, M. Kuisma, T. Korn, P. Erhart, M. Selig, and E. Malic, Interlayer exciton dynamics in van der waals heterostructures, Communications Physics **2**, 10.1038/s42005-019-0122-z (2019).

[4] A. Kormányos, G. Burkard, M. Gmitra, J. Fabian, V. Zólyomi, N. D. Drummond, and V. Fal'ko, k·p theory for two-dimensional transition metal dichalcogenide semiconductors, 2D Materials **2**, 022001 (2015).

[5] S. Latini, E. Ronca, U. De Giovannini, H. Hübener, and A. Rubio, Cavity control of excitons in two-dimensional materials, Nano Letters **19**, 3473–3479 (2019).

[6] H. Liu, F. Troisi, H. Hübener, S. Latini, and A. Rubio, Cavity-mediated electron-electron interactions: Renormalizing dirac states in graphene, arXiv 10.48550/ARXIV.2505.10166 (2025), 2505.10166.

[7] M. Ruggenthaler, N. Tancogne-Dejean, J. Flick, H. Appel, and A. Rubio, From a quantum-electrodynamical light–matter description to novel spectroscopies, Nature Reviews Chemistry **2**, 10.1038/s41570-018-0118 (2018).

[8] T. Oka and S. Kitamura, Floquet engineering of quantum materials, Annual Review of Condensed Matter Physics **10**, 387–408 (2019).

[9] S. Latini, D. Shin, S. A. Sato, C. Schäfer, U. De Giovannini, H. Hübener, and A. Rubio, The ferroelectric photo ground state of srtio$_3$: Cavity materials engineering, Proceedings of the National Academy of Sciences **118**, 10.1073/pnas.2105618118 (2021).

[10] V. Rokaj, M. Ruggenthaler, F. G. Eich, and A. Rubio, Free electron gas in cavity quantum electrodynamics, Physical Review Research **4**, 10.1103/physrevresearch.4.013012 (2022).
